# Relationship between inflammatory markers and mild cognitive impairment in Chinese patients with type 2 diabetes: a case-control study

**DOI:** 10.1186/s12902-019-0402-3

**Published:** 2019-07-11

**Authors:** Miaoyan Zheng, Baocheng Chang, Liqiang Tian, Chunyan Shan, Hui Chen, Yuxia Gao, Guowei Huang, Meilin Zhang

**Affiliations:** 1 NHC Key Laboratory of Hormones and Development, Tianjin Key Laboratory of Metabolic Diseases, Metabolic Diseases Hospital & Institute of Endocrinology, Tianjin Medical University, Tianjin, China; 2 Department of Clinical Laboratory, Tianjin Chest Hospital, Tianjin, China; 3 School of Nursing, Tianjin Medical University, Tianjin, China; 4 Department of Cardiology, Tianjin Medical University General Hospital, Tianjin, China; 5 Department of Nutrition and Food Science, School of Public Health, Tianjin Medical University, Tianjin, China; 6 Tianjin Key Laboratory of Environment, Nutrition and Public Health, Tianjin, China; 7 Center for International Collaborative Research on Environment, Nutrition and Public Health, Tianjin, China

**Keywords:** Type 2 diabetes, Mild cognitive impairment, Inflammation, Folate, High-sensitivity C-reactive protein

## Abstract

**Background:**

Several studies have indicated that inflammatory markers were associated with the risk of mild cognitive impairment (MCI) in type 2 diabetes (T2D). Serum folate was related to MCI as well as inflammation. However, no studies have investigated the association between inflammatory markers and MCI taking account of serum folate level in T2D patients. This study aimed to conduct a case-control study to evaluate the association between inflammatory markers and MCI taking account of serum folate level in Chinese patients with T2D.

**Methods:**

This study consisted of 126 T2D patients (63 cases with MCI and 63 controls without MCI). Clinical parameters, serum folate, high-sensitivity C-reactive protein (hs-CRP), interleukin-6 (IL-6), tumor necrosis factor-α (TNF-α) were measured. Spearman correlation analysis and logistic regression analysis were used to analyze the association between the inflammatory markers and the risk of MCI in T2D patients.

**Results:**

There were higher serum hs-CRP, IL-6 and TNF-α in T2D cases with MCI compared with the controls. Serum folate was negatively correlated with hs-CRP, TNF-α, and IL-6 (*P* < 0.05). In multivariate analysis, there were significant associations between serum IL-6 or hs-CRP and MCI after adjusting for the confounding variables, however, the association between hs-CRP and MCI disappeared after further adjusting for serum folate. Further subgroup analysis revealed that the significant association between hs-CRP and MCI only existed in the low folate subgroup (< 7.0 μg/L; OR = 3.34, 95% CI: 1.05–10.64), not in the high folate subgroup (≥7.0 μg/L; OR = 2.16, 95% CI: 0.68–6.88) after adjusting for the confounding variables.

**Conclusions:**

Serum IL-6 and hs-CRP were associated with the risk of MCI in Chinese patients with T2D. Serum folate might modify the association between serum hs-CRP and MCI in T2D patients.

## Background

Alzheimer disease (AD) as well as type 2 diabetes (T2D) is age-related diseases and the incidences of the two diseases are increasing at an alarming rate. Mild cognitive impairment (MCI) is characterized by an isolated memory deficit and a largely intact general cognitive functioning [1]. On the continuum of cognitive function, MCI represents a transitional state between and overlaps normal aging and AD, and it is now recognized as a risk factor for AD [2]. There have been several prospective studies reported that T2D was one of risk factors for dementia and MCI, and the relative risk of T2D to AD was approximately twice that of non-diabetics [3, 4]. Animal study [5] and human studies [6, 7] have found that diabetes could accelerate the appearance of cerebrovascular inflammation and Aβ deposition, as evidenced by increased levels of proinflammatory cytokines such as interleukin (IL-6) and tumor necrosis factor (TNF-α). Furthermore, it has been demonstrated that adequate folate might help to delay or prevention of dementia and higher folate intake might decrease the risk of AD [8]. Recent studies have shown that the serum folate was related to lower concentrations of inflammatory markers and folate intake might help to control the inflammation process [9].

In previous, one cohort study of 168 Chinese elderly patients has demonstrated that plasma high-sensitivity C-reactive protein (hs-CRP) level was associated with cognitive function and development of dementia in non-diabetics [10]. Currently, several epidemiological studies have focus on the association between inflammatory markers and the risk of MCI in T2D patients [11, 12], which showed that hs-CRP or IL-6 were found to be associated with the risk of MCI among T2D patients. Moreover, it has been reported that MCI was often associated with folate [8] and folic acid supplementation appeared to improve cognitive function and reduce blood levels of Aβ-related biomarkers in MCI [13]. To our knowledge, no studies have investigated the association between inflammatory markers and MCI taking account of serum folate level in Chinese patients with T2D. Here, we conducted this case-control study to examine the relationship between inflammatory factors and MCI in Chinese patients with T2D.

## Methods

### Subjects

**Fig. 1:**
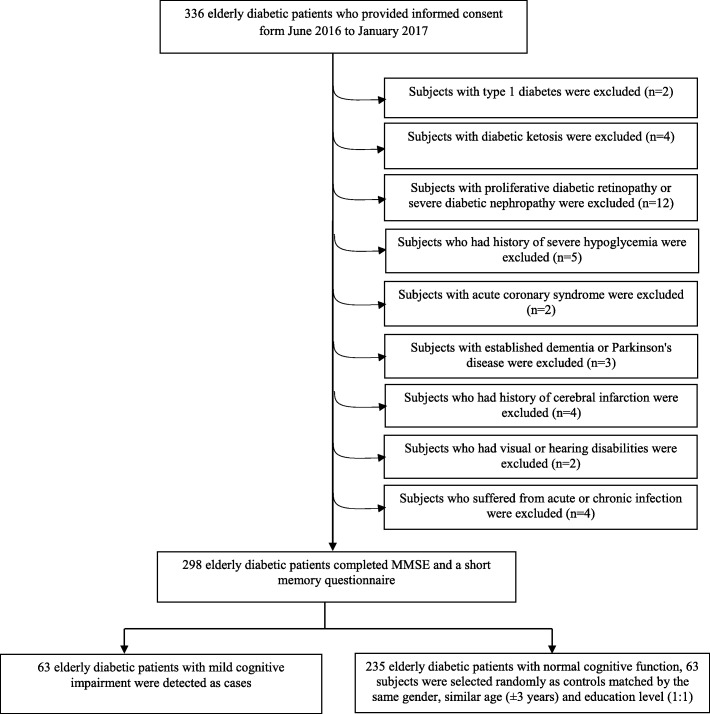
Flow chart of the sample selection process. MMSE: Mini-Mental State Examination

A case-control study was conducted at the Metabolic Disease Hospital of Tianjin Medical University from June 2016 to January 2017. The enrollment process was described in Fig. 1. Three hundred thirty-six elderly diabetic patients who were hospitalized for diabetes or its complications were enrolled. All the diabetic patients were met current international diagnostic standards for diabetes [14] and were treated with anti-diabetic medications, including oral hypoglycemic agents (OHAs) and/or insulin injection on the basis of lifestyle interventions. Subjects with hypertension were treated with antihypertensive therapy. Subjects with dyslipidemia were treated with statins, except for those with fasting serum triglyceride were treated with fibrates to prevent acute pancreatitis. Subjects were excluded from the study if they exhibited: (1) type 1 diabetes, (2) acute or severe chronic diabetic complications, such as diabetes ketosis, (3) proliferative diabetic retinopathy, diabetic foot infection or ulcer, (4) severe diabetic nephropathy: estimated glomerular filtration rate (eGFR) < 45 ml/min • 1.73m^2^; or urinary albumin creatinine ratio > 300 mg/g; or eGFR 45–60 ml/min • 1.73m^2^ and urinary albumin creatinine ratio 30-300 mg/g [15]. The eGFR was estimated by the Chronic Kidney Disease Epidemiology Collaboration creatinine equation [16], (5) history of severe hypoglycemia or hypoglycemia unawareness which the individuals required the assistance of another person to administer treatment [17], (6) acute coronary syndrome or other severe cardiovascular diseases, (7) neurological disorders, such as established dementia, Parkinson’s disease and seizures, (8) any apparent past history of a cerebrovascular events, or presence of any clinically significant abnormality in brain computed tomography or magnetic resonance imaging scan, (9) visual or hearing disabilities; psychiatric disorder, (10) acute or chronic infection, or taking NSAIDS, antibiotics or steroids within the 3 months. Altogether, 39 subjects were excluded according the above exclusion criteria. Totally 298 T2D patients completed Mini-Mental State Examination (MMSE) [18] and a short memory questionnaire [19]. Sixty-three T2D patients with MCI were detected as cases. Accordingly, among the 235 diabetic subjects with normal cognitive function (NCF), 63 T2D ones were selected randomly as controls matched by same gender, similar age (±3 years) and education using an on-line Random Number Generator/Picker according to the visiting orders of subjects. Consequently, the final sample was composed of 63 cases and 63 controls (Fig. 1).

All subjects underwent MMSE [18] and a short memory questionnaire [19] by trained interviewers. The questionnaire aimed to distinguish if the patients complained about decreased subjective memory. The MMSE is a widely used brief cognitive screening test, which assesses space/time orientation, verbal memory, language, attention/calculation, and visuoconstructive abilities. Another questionnaire was used to determine whether the subjects had any impairment in activities of daily living (ADL) [20]. The patients who met the Petersen’s diagnostic criteria [21] were defined as having MCI: subjective memory complaint and the evidence of objective memory impairment; the MMSE scores were above 13, but ≤18 or ≤ 20 or ≤ 24, with zero, or 1 to 6 years, or more than 6 years education, respectively; intact or minimal damage of ADL; but failure to meet the DSM-IIIR criteria of dementia [22]. The participants with NCF were defined as the subjects who had no subjective memory complaint or objective memory damage, and MMSE scores over the above values based on the corresponding education level.

The study protocol was approved by the Ethics Committee of Tianjin Medical University. Written informed consent was received from each patient.

### Data collection

Height was measured without shoes to the nearest 1 cm and weight measured in light clothing to the nearest 0.1 kg on a beam balance scale. Body mass index (BMI) was calculated as weight in kilograms divided by the square of the height in meters (kg/m^2^). Blood pressure was measured twice on the left arm with subjects in a seated position after 15 min rest. These measures were averaged to get a mean blood pressure. Information about demographic characteristics, medication history, duration of diabetes, anti-diabetic medications (including OHAs and/or insulin) and diabetic complications, as well as lifestyle habits (smoking, drinking and physical activity) were collected by trained interviewers. Venous blood samples were taken from all participants after an overnight fast (12 h at least) and the samples were stored at − 80 °C until assessment assayed performed. The glycated hemoglobin A1c (GHbA1c) was measured using high-performance liquid chromatography method. The levels of lipid profile including total cholesterol (TC), triglycerides (TG), high-density lipoprotein cholesterol (HDL-C), low-density lipoprotein cholesterol (LDL-C) and fasting plasma glucose (FPG) were measured enzymatically on a Modular Analytics P800 analyzer (Roche Diagnostics, Indianapolis, IN).

### Measurement of serum folate and inflammation markers

Blood samples (2 mL) were collected into anticoagulant-free tubes and centrifuged at 1000×*g* for 15 min. Serum folate was determined by chemiluminescent assay (Access Immuno Systems, Beckman Coulter, Brea, CA). Serum hs-CRP was determined by immunologic turbidimetry (Orion Diagnostic, Espoo, Finland). Serum levels of TNF-α and IL-6 were measured in duplicate using a commercially available enzyme-linked immunosorbent assay kit (Human IL-6 and Quantikine HS TNF-α, Boster Biological Technology, Wuhan, China).

### Statistical analysis

Mean and standard deviations (SD) or median (inter-quartile range) were used as descriptive statistics for continuous variables and percentages for categorical variables. For continuous variables, the paired-sample *t* test or Wilcoxon rank-sum test was used for between-group comparisons, and chi-square test for categorical variables. Spearman correlation analysis was conducted to analyze the correlation between serum folate and inflammatory markers. Logistic regression analysis was used to assess the association between the inflammatory markers and risk of MCI. Odds ratio (OR) and corresponding 95% confidence intervals (CI) were calculated. Model 1 was used to calculate the crude OR, and model 2 was adjusted for factors related diabetes including duration of diabetes (per year), HbA1c (< 7.0%/≥7.0%), anti-diabetic medications (only OHAs /only insulin/insulin plus OHAs), diabetic retinopathy (yes/no) and diabetic nephropathy (yes/no), and common cardiovascular diseases risk factors including smoking status (yes/no), body mass index (< 24.0 kg/m^2^/24.0–28.0 kg/m^2^/≥28.0 kg/m^2^), hypertension (yes/no), LDL-C (< 2.6 mmol/L/≥2.6 mmol/L). Model 3 was additional adjusted for serum folate. All statistical operations were performed using SPSS (version 13.0, Chicago, IL, USA). All reported *P* values were two-sided and *P* value less than 0.05 was considered to indicate a statistically significant difference.

## Results

### Characteristics of study samples

The socioeconomic data and MMSE scores of the subjects were shown in Table 1. Compared with the subjects in T2D-NCF group, those in T2D-MCI group had lower MMSE scores (20.38 ± 3.68 vs. 26.75 ± 2.84; *P* < 0.001). The mean level of TC (5.45 ± 1.10 mmol/L) in T2D-MCI group was significantly higher than that in T2D-NCF group (5.00 ± 1.00 mmol/L, *P* < 0.05). The percentage of diabetic retinopathy (42.9%), diabetic nephropathy (41.3%), LDL-C ≥ 2.60 mmol/L (84.1%) in T2D-MCI group were significantly higher than that in T2D-NCF group (19.0, 19.0 and 66.7%, respectively; *P* < 0.05). There were no significant differences in BMI, systolic blood pressure (SBP), diastolic blood pressure (DBP), the duration of diabetes, triglycerides, HDL-C, FPG, GHbA1c, the percent of hypertension, ADL scores, smoking status and drinking status between T2D-MCI group and T2D-NCF group (*P* > 0.05). In addition, there were no significant differences in all the above indices between the low folate subgroup (< 7.0 μg/L) and the high folate subgroup (≥7.0 μg/L) in T2D-MCI group or T2D-NCF group (*P* > 0.05) (Table 1).

**Table 1:** Characteristics according to cognitive status

Variables	T2D-NCF group			T2D-MCI group			*P* value^a^
	Low folate (< 7.0 μg/L) (*n* = 23)	High folate (≥7.0 μg/L) (*n* = 40)	Total (*n* = 63)	Low folate (< 7.0 μg/L) (*n* = 38)	High folate (≥7.0 μg/L) (*n* = 25)	Total (*n* = 63)	
Sex							
Men	9 (39.1)	13 (32.5)	22 (34.9)	17 (44.7)	5 (20.0)	22 (34.9)	> 0.999
Women	14 (60.9)	27 (67.5)	41 (65.1)	21 (55.3)	20 (80.0)	41 (65.1)	
Education							
No education	5 (21.7)	8 (20.0)	13 (20.6)	7 (18.4)	6 (24.0)	13 (20.6)	> 0.999
1–6 years	5 (21.8)	10 (25.0)	15 (23.8)	10 (26.3)	5 (20.0)	15 (23.8)	
≥6 years	13 (56.5)	22 (55.0)	35 (55.6)	21 (55.3)	14 (56.0)	35 (55.6)	
Age (years)	73.70 ± 3.10	74.28 ± 4.78	74.06 ± 4.22	74.55 ± 4.17	74.84 ± 5.01	74.67 ± 4.48	0.148
MMSE	26.65 ± 2.89	26.80 ± 2.84	26.75 ± 2.84	20.68 ± 3.99	19.92 ± 3.73	20.38 ± 3.68	< 0.001
ADL	21.30 ± 1.85	22.13 ± 2.96	21.83 ± 2.62	22.24 ± 3.22	23.00 ± 3.35	22.54 ± 3.27	0.217
Smoking status							
Yes	10 (43.5)	11 (27.5)	21 (33.3)	17 (44.7)	8 (32.0)	25 (39.7)	0.459
No	13 (56.5)	29 (72.5)	42 (66.7)	21 (55.3)	17 (68.0)	38 (60.3)	
Drinking status							
Yes	5 (21.7)	9 (22.5)	14 (22.2)	7 (18.4)	3 (12.0)	10 (15.9)	0.364
No	18 (78.3)	31 (77.5)	49 (77.8)	31 (81.6)	22 (88.0)	53 (84.1)	
Body mass index (kg/m^2^)	26.03 ± 3.78	25.71 ± 2.85	25.82 ± 3.19	26.41 ± 3.99	26.43 ± 4.07	26.42 ± 4.00	0.377
Body mass index							
< 24.0 kg/m^2^	7 (30.4)	11 (27.5)	18 (28.6)	10 (26.3)	9 (36.0)	19 (30.2)	0.749
24.0–28.0 kg/m^2^	8 (34.8)	19 (47.5)	27 (42.8)	16 (42.1)	7 (28.0)	23 (36.5)	
≥28.0 kg/m^2^	8 (34.8)	10 (25.0)	18 (28.6)	12 (31.6)	9 (36.0)	21 (33.3)	
Systolic blood pressure (mmHg)	137.17 ± 16.98	131.13 ± 18.86	133.33 ± 18.29	136.71 ± 16.78	131.20 ± 11.75	134.52 ± 15.13	0.677
Diastolic blood pressure (mmHg)	76.96 ± 10.53	73.88 ± 8.59	75.00 ± 9.38	77.24 ± 8.60	74.40 ± 6.67	76.11 ± 7.95	0.475
Diabetes Duration (years)	15.0 (8.0, 20.0)	16.0 (9.3, 21.5)	16.0 (9.0, 20.0)	14.5 (9.0, 20.0)	15.0 (10.0, 22.0)	15.0 (9.0, 20.0)	0.833
Anti-diabetic medications							
OHAs	7 (30.4)	10 (25.0)	17 (27.0)	7 (18.4)	7 (28.0)	14 (22.2)	0.799
Insulin	5 (21.7)	8 (20.0)	13 (20.6)	10 (26.3)	5 (20.0)	15 (23.8)	
Insulin + OHAs	11 (47.8)	22 (55.0)	33 (52.4)	21 (55.3)	13 (52.0)	34 (54.0)	
Triglycerides (mmol/L)	1.27 (0.98, 1.94)	1.33 (0.97, 2.18)	1.32 (0.98, 2.01)	1.75 (1.14, 2.22)	1.43 (1.19, 2.48)	1.71 (1.15, 2.28)	0.135
Total cholesterol (mmol/L)	5.06 ± 0.99	4.97 ± 1.03	5.00 ± 1.00	5.27 ± 1.19	5.73 ± 0.91	5.45 ± 1.10	0.013
HDL-C (mmol/L)	1.41 ± 0.40	1.39 ± 0.32	1.40 ± 0.35	1.31 ± 0.27	1.42 ± 0.30	1.36 ± 0.29	0.483
LDL-C (mmol/L)							
< 2.6 mmol/L	8 (34.8)	13 (32.5)	21 (33.3)	6 (15.8)	4 (16.0)	10 (15.9)	0.023
≥2.6 mmol/L	15 (65.2)	27 (67.5)	42 (66.7)	32 (84.2)	21 (84.0)	53 (84.1)	
Fasting plasma glucose (mmol/L)	9.03 ± 3.53	7.80 ± 1.74	8.25 ± 2.59	8.62 ± 2.58	9.18 ± 3.00	8.74 ± 2.72	0.338
Glycated hemoglobin A1c (%)	8.70 ± 2.53	7.95 ± 1.51	8.22 ± 1.96	8.34 ± 2.02	9.19 ± 2.64	8.68 ± 2.31	0.220
< 7.0%	5 (21.7)	14 (35.0)	19 (30.2)	6 (15.8)	4 (16.0)	10 (15.9)	0.057
≥7.0%	18 (78.3)	26 (65.0)	44 (69.8)	32 (84.2)	21 (84.0)	53 (84.1)	
Hypertension							
Yes	19 (82.6)	23 (57.5)	42 (66.7)	28 (73.7)	18 (72.0)	48 (73.0)	0.437
No	4 (17.4)	17 (42.5)	21 (33.3)	10 (26.3)	7 (28.0)	17 (27.0)	
Diabetic retinopathy							
Yes	4 (17.4)	8 (20.0)	12 (19.0)	16 (42.1)	11 (44.0)	27 (42.9)	0.004
No	19 (82.6)	32 (80.0)	51 (81.0)	22 (57.9)	14 (56.0)	36 (57.1)	
Diabetic nephropathy							
Yes	4 (17.4)	8 (20.0)	12 (19.0)	15 (39.5)	11 (44.0)	26 (41.3)	0.007
No	19 (82.6)	32 (80.0)	51 (81.0)	23 (60.5)	14 (56.0)	37 (58.7)	

Data are means ± standard deviation, median (inter-quartile range), or n (%)

*T2D-NCF* Type 2 diabetes with normal cognitive function, *T2D-MCI* Type 2 diabetes with mild cognitive impairment, *ADL* Activities of daily living, *MMSE* Mini-mental state examination, *OHAs* Oral hypoglycemic agents, *HDL-C* High-density lipoprotein cholesterol, *LDL-C* Low-density lipoprotein cholesterol

^a^ *P* value was are significant difference between normal cognitive function group and mild cognitive impairment group

### Comparisions of serum folate and inflammatory markers according to cognitive status

The median value of serum folate in T2D-MCI group was significantly lower than that in T2D-NCF group (T2D-MCI: 6.25(5.30–7.94) μg/L vs. T2D-NCF: 8.07(6.65–10.40) μg/L) (*P* < 0.01) (Table 2). Compared with the T2D-NCF group, the median values of serum TNF-α, IL-6 and hs-CRP were significantly higher than that in T2D-MCI group (*P* < 0.05) (Table 2).

**Table 2:** Comparisions of serum folate and IL-6, TNF-α, hs-CRP according to cognitive status

Variables	T2D-NCF group (*n* = 63)	T2D-MCI group (*n* = 63)	*P* value
Folate (μg/L)	8.07 (6.65, 10.40)	6.25 (5.30, 7.94)	< 0.001
TNF-α (pg/ml)	28.95 (22.94, 34.26)	32.02 (26.32, 38.47)	0.013
IL-6(pg/ml)	16.94 (14.79, 22.75)	25.62 (20.80, 28.71)	< 0.001
hs-CRP (mg/L)	0.80 (0.50, 1.50)	1.40 (0.80, 3.10)	< 0.001

Values represent the median with inter-quartile range

*T2D-NCF* Type 2 diabetes with normal cognitive function, *T2D-MCI* Type 2 diabetes with mild cognitive impairment, *IL-6* Interleukin-6, *TNF-α* Tumor necrosis factor-α, *hs-CRP* High-sensitivity C-reactive protein

### The correlations between serum folate and inflammatory markers

Spearman correlation analysis showed that the serum folate was negatively correlated with hs-CRP, TNF-α, and IL-6 (*r* value: − 0.33, − 0.19, − 0.21, respectively; *P* < 0.05) (Table 3).

**Table 3:** The correlations between serum folate and inflammatory markers

Inflammatory markers	Folate (μg/L)	
	*r* value	*P* value
TNF-α (pg/ml)	−0.19	0.031
IL-6(pg/ml)	−0.21	0.019
hs-CRP (mg/L)	−0.33	< 0.001

*TNF-α* Tumor necrosis factor-α, *IL-6* Interleukin-6, *hs-CRP* High-sensitivity C-reactive protein

### Odds ratios of MCI for inflammatory markers

Table 4 showed the odds ratios of MCI across tertiles of inflammatory markers. In univariate analysis, compared with the subjects in the lowest tertile, those in the upper tertile of IL-6 (OR = 3.09, 95%CI: 1.57–6.10; *P* < 0.05) and hs-CRP (OR = 2.25, 95%CI: 1.14–4.44; *P* < 0.05) were associated with higher odds of MCI. The TNF-α was not associated with risk of MCI (*P* > 0.05). In multivariate analysis, the significant associations persisted after adjusted for the confounding variables including smoking, overweigh and obesity, duration of diabetes, anti-diabetic medications, HbA1c, hypertension, LDL-C, diabetic retinopathy and diabetic nephropathy. The significant association between IL-6 and MCI persisted after further adjusting for serum folate, whereas the association between hs-CRP and MCI disappeared. Since the greatest reduction in neural tube defects has been observed at serum concentrations≥7.0 μg/L [23], we further divided all the subjects into two subgroups according to the level of serum folate (< 7.0 μg/L/≥7.0 μg/L) and evaluated the associations between hs-CRP and MCI among the two subgroups (Table 5). The significant association between hs-CRP and MCI only existed in the low folate subgroup (< 7.0 μg/L) after adjusted for the above confounding factors (OR = 3.34, 95%CI: 1.05–10.64), not in the high folate subgroup (≥7.0 μg/L; OR = 2.16, 95% CI: 0.68–6.88).

**Table 4:** Odds ratios of MCI for inflammatory markers

Variables	Tertile	Controls/ Case	Model 1		Model 2		Model 3	
			OR(95%CI)	*P* value	OR(95%CI)	*P* value	OR(95%CI)	*P* value
TNF-α	< 26.33 pg/ml	26/16	1.00	–	1.00	–	1.00	–
	26.33–33.83 pg/ml	20/22	1.38 (0.73–2.62)	0.332	1.46 (0.75–2.83)	0.133	1.48 (0.76–2.89)	0.250
	≥33.83 pg/ml	17/25	1.56 (0.83–2.93)	0.163	1.47 (0.77–2.80)	0.238	1.41 (0.74–2.69)	0.296
IL-6	< 16.91 pg/ml	31/11	1.00	–	1.00	–	1.00	–
	16.91–25.35 pg/ml	24/18	1.64 (0.77–3.47)	0.198	1.90 (0.88–4.12)	0.105	1.96 (0.90–4.26)	0.091
	≥25.35 pg/ml	8/34	3.09 (1.57–6.10)	0.001	3.15 (1.58–6.28)	0.001	3.12 (1.56–6.24)	0.001
hs-CRP	< 0.63 mg/L	30/12	1.00	–	1.00	–	1.00	–
	0.63–1.67 mg/L	18/24	2.00 (1.00–4.00)	0.050	1.87 (0.92–3.79)	0.083	1.79 (0.88–3.66)	0.108
	≥1.67 mg/L	15/27	2.25 (1.14–4.44)	0.019	2.14 (1.05–4.35)	0.035	2.02 (0.98–4.14)	0.055

Model 1: Crude model

Model 2: Adjusted for diabetes related factors including duration of diabetes (per year), HbA1c (< 7.0%/≥7.0%), anti-diabetic medications (only oral hypoglycemic agents /only insulin/insulin plus oral hypoglycemic agents), diabetic retinopathy (yes/no) and diabetic nephropathy (yes/no), and common cardiovascular diseases risk factors including smoking status (yes/no), body mass index (< 24.0 kg/m^2^/24.0–28.0 kg/m^2^/≥28.0 kg/m^2^), hypertension (yes/no), LDL-C (< 2.6 mmol/L/≥2.6 mmol/L)

Model 3: Model 2 plus serum folate (< 7.0 μg/L/≥7.0 μg/L)

*IL-6* Interleukin-6, *TNF-α* Tumor necrosis factor-α, *hs-CRP* High-sensitivity C-reactive protein

**Table 5:** Odds ratios of MCI for inflammatory markers stratified by serum folate ^a^

Variable	Tertile	Low folate subgroup(< 7.0 μg/L) (*n* = 61)			High folate subgroup(≥7.0 μg/L) (*n* = 65)		
		Controls/ Case	OR(95%CI)	*P* value	Controls / Case	OR(95%CI)	*P* value
hs-CRP	< 0.63 mg/L	12/4	1.00	–	18/8	1.00	–
	0.63–1.67 mg/L	6/15	2.95 (0.90–9.63)	0.074	12/9	2.04 (0.68–6.17)	0.205
	≥1.67 mg/L	5/19	3.34 (1.05–10.64)	0.042	10/8	2.16 (0.68–6.88)	0.194

^a^ Adjusted for diabetes related factors including duration of diabetes (per year), HbA1c (< 7.0%/≥7.0%), anti-diabetic medications (only oral hypoglycemic agents /only insulin/insulin plus oral hypoglycemic agents), diabetic retinopathy (yes/no) and diabetic nephropathy (yes/no), and common cardiovascular diseases risk factors including smoking status (yes/no), body mass index (< 24.0 kg/m^2^/24.0–28.0 kg/m^2^/≥28.0 kg/m^2^), hypertension (yes/no), LDL-C (< 2.6 mmol/L/≥2.6 mmol/L)

## Discussion

Since AD and MCI is age-related disease and male sex as well as less education is significantly associated with cognitive decline in stroke-free patients, and some inflammatory markers such as TNF-α and IL-6 levels increase with increasing age in humans [24], we randomly selected the cases and controls matched by the same gender, similar age (±3 years) and education level. Several related factors of diabetes and its major complications, as well as cardiovascular diseases risk factors were correlated with inflammation [25–27] or cognition [3], we have excluded the patients who suffered cerebrovascular events or acute coronary syndrome. Intensive hypoglycemic therapy has been proposed to reduce the risk of diabetic complications. However, the insulin or sulfonylureas might increase the risk of severe hypoglycaemia, which was associated with an increased risk of cognitive dysfunction or dementia [28]. Thus, the subjects with history of severe hypoglycemia due to overdose of insulin or sulfonylureas were also excluded. The major finding of this study was that the T2D patients with MCI had higher plasma hs-CRP, IL-6 and TNF-α compared with the T2D patients with normal cognition. There was significant association between serum IL-6 or hs-CRP and MCI after adjusting for common confounding factors, however, the association between hs-CRP and MCI disappeared after further adjusting for serum folate.

Numerous studies have indicated the association of inflammation markers with increased risks for dementia, MCI, and cognitive decline [6, 29, 30]. Zuliani G et al. have demonstrated that high levels of IL-1β and TNF-α, but not IL-6, were associated with increased likelihood of having late-onset AD [31]. Similarly, Tarkowski et al. have reported that the TNF-a, but not IL-6 level was significantly higher in cerebrospinal fluid of AD patients [32]. In contrast, Schuitemaker et al. [33] have reported that serum IL-6 level was significantly higher in MCI, which suggested that inflammatory processes might be involved in early stages of AD, and different inflammatory markers might play major roles in certain developing stages of cognition decline. Moreover, Takeda et al. have found that IL-6 and TNF-α were significantly higher in the brain microvessels of a new mice model that reflects the pathological conditions of both AD and diabetes compared with other genotypes which only reflects unique pathological characteristics of AD or diabetes. This indicated diabetic condition might enhance cognitive dysfunction by aggravating inflammation [5]. Our findings were similar to a cross-sectional study from Scotland [12], which has shown that IL-6 was associated with estimated lifetime cognitive status after adjusting for vocabulary, education level, cardiovascular dysfunction, duration of diabetes, and glycemic control in older patients with T2D. The findings of a recent study from China have also suggested that plasma hs-CRP was associated with the risk of MCI among T2D patients [11]. Meanwhile, another Chinese case-control study in T2D has reported that the patients with MCI have a significantly higher hs-CRP than those without MCI, but CRP was not significantly correlated with the cognitive status [34]. Since aging was associated with chronic low-grade increases in circulating levels of inflammatory markers [24], the results differed from ours might due to the younger subjects (aged 63.45 ± 8.38 years) than ours (74.37 ± 4.35 years). Recently, a meta-analysis has found that IL-6 level was inversely correlated with mean MMSE scores among numerous of peripheral inflammatory markers in patients with AD [7]. Plasma IL-6 level was inversely with hippocampal grey matter volume, which was a structure critical for memory formation and associated with cognition decline [35]. Our findings were consistent with the above studies, which suggested that the inflammatory cytokine, IL-6 and hs-CRP might be a meaningful biological marker to link the cognitive impairment in T2D patients.

Our findings was consistent with the observation that inverse associations between serum hs-CRP and folate concentration existed in pregnant women [9]. A recent cross-sectional study of adolescents in Sweden has demonstrated that serum hs-CRP was significantly inversely associated with folate intakes [36]. As we known, hs-CRP is a reactant involved in the acute-phase response and rapidly increases in the presence of infection or inflammation, which is stimulated by the release of proinflammatory cytokines, including IL-1, IL-6 and TNF-α. Our study has suggested that folate might help control the acute-phase reactions of inflammation in T2D patients with MCI.

We further included serum folate in the model to assess whether the associations between inflammation markers and MCI were mediated by serum folate level. Considering the MCI could be directly linked to the inflammation markers or the serum folate might have an indirect effect on MCI through improving the inflammation markers, we evaluated the associations of inflammation markers and MCI after further adjusting for serum folate. In the present study, there was a significant positive association of serum hs-CRP and MCI after adjusted for common diabetes related factors and cardiovascular diseases risk factors among T2D patients in the low folate subgroup (< 7.0 μg/L). Our results have indicated that there might be somewhat relationship among peripheral folate, inflammatory cytokines and cognition status [37]. It has been reported that folate deficiency might enhance the expression of the inflammatory mediators including IL-1β, IL-6 and TNF-α, independent of the concentration of homocysteine [38]. On the contrary, folic acid may inhibit lipopolysaccharide-induced production of IL-1β and TNF-α through inhibiting the MAPKs and NF-κB pathways [39]. A recent animal study aimed to evaluate the efficacy of folic acid in prevention of apoptosis by inhibiting TNF-α action in ischemia-reperfusion induced liver injury has also shown that folic acid might inhibit apoptosis by inhibiting the action of TNF-α [40]. Moreover, folic acid supplement to the patients with MCI could significantly reduce peripheral TNF-α and IL-6 levels [41]. These data indicated that optimal serum folate status might influence the release of proinflammatory cytokines such as IL-6 and TNF-α among T2D patients with MCI. Thus, more randomized controlled trials will be performed to explore whether serum folate might have an indirect effect on MCI through improving the inflammation reactions among T2D patients.

The previous studies have demonstrated that low folate and elevated homocysteine levels were associated with MCI in older population [42]. Low folate status, which is a well-known strong determinant of homocysteine levels, could lead to elevated homocysteine, which in turn lead to cognitive impairment [43]. Moreover, other nutrients other than folate or dietary pattern may be exert greater effects on inflammatory processes and neurodegeneration. For instance, the Mediterranean diet (MD) rich in polyunsaturated fatty acids, trace elements and vitamins, has shown promising associations with slower rate of cognitive decline [44] due to its proven anti-inflammatory [45]. Thus, the serum homocysteine and other nutrients such as vitamin C, vitamin E, vitamin B_12_ and vitamin D will be measured to explore the effects of other related nutrients on the relationship between inflammatory markers and MCI in T2D patients in future study. On the other hand, the relationship between inflammation and MCI did not exist among the subjects with high folate (≥7.0 μg/L) in our study, which indicated that there might be some other main factors to link inflammation and MCI. In our study, the patients with mild microvascular complications such as persistent microalbuminuria, non-proliferative diabetic retinopathy were enrolled. And the percentage of diabetic retinopathy (42.9%) and diabetic nephropathy (41.3%) in the case group was significantly higher than that in the control group (19.0 and 19.0%, respectively). The persistent albuminuria or diabetic retinopathy was associated with a decline in cognitive function with diabetes [46, 47]. And both diabetic nephropathy and retinopathy were vascular risk factors. One possible mechanism of MCI related to diabetic microvascular complication was that the decrease of nitric oxide in the patients with diabetic retinopathy and diabetic nephropathy [48, 49], which could lead to vascular impairment due to endothelial cell injury. In addition, the plasma levels of inflammatory biomarkers were associated with the incidence and development of diabetic vascular complication in type 1 and type 2 diabetes [50]. Thus, the vascular factors might be considered as a main factor that links inflammation and MCI in the high folate subgroup.

The following limitations of the current study should be considered. First, case-control design, temporality is not clear; further longitudinal studies and randomized controlled trials are needed to examine the temporal sequence of this association. Second, the results of this case-control study may be misinterpreted because of the influence of farraginous factors, random and systematic recall errors, and selection bias. Third, the small sample size and single ethnicity of the surveyed subjects may limit the application of our results to other ethnic groups. Finally, the conclusion of this study should be interpreted carefully as T2DM itself may increase the risk of MCI via different mechanisms.

## Conclusions

Serum IL-6 and hs-CRP were associated with the risk of MCI among Chinese patients with T2D. Moreover, serum hs-CRP was associated with MCI only among T2D patients with low serum folate level (< 7.0 μg/L), but not among those whose serum folate level was higher than 7.0 μg/L. It suggested that serum folate levels might modify the association between serum hs-CRP and MCI.

## Data Availability

The data that support the findings of this study are available on request from the corresponding author on reasonable request.

## Abbreviations

AD: Alzheimer disease
ADL: Activities of daily living
BMI: Body mass index
CI: Confidence intervals
DBP: Diastolic blood pressure
eGFR: Estimated glomerular filtration rate
FPG: Fasting plasma glucose
GHbA1c: Glycated hemoglobin A1c
HDL-C: High-density lipoprotein cholesterol
hs-CRP: High-sensitivity C-reactive protein
IL-6: Interleukin-6
LDL-C: Low-density lipoprotein cholesterol
MCI: Mild cognitive impairment
MMSE: Mini-Mental State Examination
NCF: Normal cognitive function
OHAs: Oral hypoglycemic agents
OR: Odds ratio
SBP: Systolic blood pressure
T2D: Type 2 diabetes
T2D-MCI: Type 2 diabetes with mild cognitive impairment
T2D-NCF: Type 2 diabetes with normal cognitive function
TC: Total cholesterol
TG: Triglycerides
TNF-α: Tumor necrosis factor-α
